# The Complete Chloroplast Genomes of Two *Lancea* Species with Comparative Analysis

**DOI:** 10.3390/molecules23030602

**Published:** 2018-03-07

**Authors:** Xiaofeng Chi, Jiuli Wang, Qingbo Gao, Faqi Zhang, Shilong Chen

**Affiliations:** 1 Key Laboratory of Adaptation and Evolution of Plateau Biota, Northwest Institute of Plateau Biology, Chinese Academy of Sciences, Xining 810001, China; xfchi@nwipb.cas.cn (X.C.); wang_jiul@163.com (J.W.); qbgao@nwipb.cas.cn (Q.G.); 2 University of Chinese Academy of Sciences, Beijing 100049, China; 3 Qinghai Provincial Key Laboratory of Crop Molecular Breeding, Xining 810001, China

**Keywords:** Mazaceae, Laminales, organellar genome, phylogenetic analysis

## Abstract

The genus *Lancea* is native to the Qinghai-Tibetan Plateau and consists of two species, *Lancea tibetica* Hook. f. et Thoms. and *Lancea hirsuta* Bonati. Here, we report the complete sequences of the chloroplast genomes of *L. tibetica* and *L. hirsuta*, which were 153,665 and 154,045 bp in length, respectively, and each included a pair of inverted repeated regions (25,624 and 25,838 bp in length, respectively) that were separated by a large single copy region (84,401 and 84,588 bp in length, respectively) and a smaller single copy region (18,016 and 17,781 bp in length, respectively). A total of 106 genes in *L. tibetica* and 105 in *L. hirsuta* comprised 79 protein-coding genes, and 4 ribosomal RNA (rRNA) genes, as well as 23 and 22 transfer RNA (tRNA) genes in *L. tibetica* and *L. hirsuta*, respectively. The gene order, content, and orientation of the two *Lancea* chloroplast genomes exhibited high similarity. A large number of informative repetitive sequences, including SSRs, were observed in both genomes. Comparisons of the genomes with those of three other Lamiales species revealed 12 highly divergent regions in the intergenic spacers and in the *matK*, *rpoA*, *rps19*, *ndhF*, *ccsA*, *ndhD*, and *ycf1* coding regions. A phylogenomic analysis suggested that *Lancea* forms a monophyletic group that is closely related to the clade composed of the families Phrymaceae, Paulowniaceae, and Rehmanniaceae.

## 1. Introduction

Chloroplasts, which originated from ancient endosymbiotic cyanobacteria, are specialized photosynthetic organelles for photosynthesis and carbon fixation as well as fatty acid synthesis, amino acid synthesis, and the immune response in plants [1]. Chloroplasts also possess their own genomes and genetic systems. Most chloroplast genomes range from 120 to 160 kb in length and have a typical quadripartite structure with two copies of inverted repeats (IRs) separating the large single copy (LSC) region and the small single copy (SSC) region [2]. Recently, lots of fragments from chloroplast genomes such as *rbcL* and *matK* have been widely used in plant systematics research due to its maternal inheritance and highly conserved structures [3]. With the reduction of sequencing cost, the complete chloroplast genome sequences are becoming an increasingly used and effective tool in the study of plant phylogenetic classification, molecular identification and genetic diversity [4]. The comparative analysis of chloroplast genomes is especially useful for inferring new and important insights to resolve many enigmatic phylogenetic relationships for their relatively stable genome structure, gene content, and gene order.

*Lancea* is a small genus consisting of two species, *Lancea tibetica* Hook. f. et Thoms. and *Lancea hirsuta* Bonati, both of which are native to the Qinghai-Tibetan Plateau. The main morphological differences between *L. tibetica* and *L. hirsuta* are their respective absence and presence of coarse multicellular hairs on their stems and leaves [5]. *L. tibetica* is found along streams in grasslands and sparse forests at an approximate altitude of 2000–4500 m above sea level in Gansu, Qinghai, Sichuan, and the Tibet region of China as well as Bhutan, India, and Nepal. However *L. hirsuta* is exclusively endemic to northwest Sichuan, northwest Yunnan, southeast Qinghai and the Tibet region of China [6]. *L. tibetica* also is an important traditional Tibetan medicine that has been used in the treatment of leukemia, intestinal angina, heart disease, and coughs [7,8,9].

The systematic position of *Lancea* has been debated for years. Wettstein (1891) placed *Mazus*, *Dodartia*, and *Lancea* in the tribe Gratioleae, subtribe Mimulinae based on their morphological traits [10]. However, recent molecular studies have indicated that the Scrophulariaceae are not monophyletic [11]. *Lancea* was then transferred to Phrymaceae according to the phylogenetic analysis of chloroplast *trnL/F* and nuclear ribosomal ITS and ETS sequence data [12]. However, no support was found for the monophyly of Phrymaceae. The two subfamilies of Phrymaceae-Phrymoideae and Mazoideae do not form a monophyletic clade in any of the trees in different studies [13]. Consequently, the Angiosperm Phylogeny Group IV (APG IV) classification system separated *Lancea*, *Mazus*, and *Dodartia* from Phrymaceae, placing them into a new family named Mazaceae, which is the outgroup to Paulowniaceae and Orobanchaceae [14]. Although chloroplast and/or nuclear DNA data provide some information about the taxonomy of *Lancea*, different phylogenetic relationships were inferred when phylogenies were constructed using different sequence fragments, thus requiring confirmation by the use of comprehensive genomic data [11,12,13,15,16,17]. Until now, comparative genomics approaches to the study of genetic diversity and phylogenetics in Mazaceae has been limited.

In the present study, we report the complete chloroplast genomes of *L. tibetica* and *L. hirsuta*, which were derived using next-generation sequencing. To our knowledge, this is the first report on complete chloroplast genome in the Mazaceae family. Genomic information about *L. tibetica* and *L. hirsuta* is fundamental to supporting current conservation efforts especially phylogenetic analysis of these rare species. Our aim was to compare the full chloroplast genomes of these two species, which will serve as valuable genomic resources.

## 2. Materials and Methods

### 2.1. DNA Extraction and Sequencing

*L. tibetica* was sampled from a single plant collected in Qumalai (96°34′38.8′′ E, 33°58′03.1′′ N, Qinghai, China), while a single sample of *L. hirsuta* was collected from Zaduo (95°00′16′′ E, 32°51′51′′ N, Qinghai, China). The DNA of the two species was isolated from fresh leaves via the modified CTAB method [18]. The DNA content was measured using a NanoDrop spectrophotometer (Thermo Scientific, Carlsbad, CA, USA). Each DNA sample was randomly fragmented to construct paired-end libraries according to the Illumina preparation manual (San Diego, CA, USA). We sequenced the complete chloroplast genomes using the Illumina MiSeq platform at Novogene Biotech Co. (Beijing, China).

### 2.2. Chloroplast Genome Assembly and Annotation

Genomic sequences were screened and assembled with SOAPdenovo [19]. To test the assemble accuracy around IR-LSC/SSC junctions, four primers listed in Table S1 were used to amplify the junctions of IRs and the LSC/SSC. These PCR products were analyzed via Sanger sequencing using the primers mentioned above. Annotation was performed with CpGAVAS (http://www.herbalgenomics.org/cpgavas) [20] coupled with manual adjustment of start/stop codons and intron/exon borders after BLAST searches. The gene homologies were confirmed by comparing them with NCBI’s non-redundant (Nr) protein database, Clusters of orthologous groups for eukaryotic complete genomes (KOG), KEGG (http://www.kegg.jp/), GO (http://www.geneontology.org), PFAM (http://xfam.org), SWISS-PROT (http://web.expasy.org/docs/swissprotguideline.html), and TREMBL (http://www.bioinfo.pte.hu/more/TrEMBL.htm) databases. TRNAscan-SE 1.21 were introduced to confirm the transfer RNAs (tRNAs). The circular maps of the two *Lancea* chloroplast genomes were drawn with OrganellarGenomeDRAW (OGDRAW; http://ogdraw.mpimp-golm.mpg.de/index.shtml) [21]. The annotated genomic sequences have been submitted to GenBank under accession numbers MF593117 and MG551489 for *L. tibetica* and *L. hirsuta*, respectively.

### 2.3. Repeat Structure Analysis

Dispersed and palindromic repeats were identified by the REPuter program (http://bibiserv2.cebitec.uni-bielefeld.de/reputer) [22]. The minimal size was set to 30 bp with >90% identity (Hamming distance equal to 3) between the two repeats. MSDB 2.4 (https://code.google.com/archive/p/msdb/downloads) [23] was used to detect simple sequence repeats (SSRs) with minimal repeat numbers of 10, 5, 4, 3, 3, and 3 for mono-, di-, tri-, tetra-, penta-, and hexa-nucleotides, respectively.

### 2.4. Genome Comparison

The mVISTA program (http://genome.lbl.gov/vista/mvista/about.shtml) [24] was employed in Shuffle-LAGAN mode to determine differences in the chloroplast genomes of *L. tibetica* and *L. hirsuta* with those of *Rehmannia chingii* (KX426347), *Paulownia coreana* (KP718622), and *Erythranthe lutea* (KU705476), all of which were obtained from GenBank. The nucleotide variability (average pairwise divergence) between the *L. tibetica* and *L. hirsuta* chloroplast genomes was calculated using DnaSP v5.10 (http://www.ub.edu/dnasp/DnaSP_OS.html) [25] with a sliding window analysis. Window length was set to 400 bp, and the step size was 200 bp.

### 2.5. Phylogenetic Analysis

Phylogenetic analysis was performed among *L. tibetica*, *L. hirsuta*, and 21 outgroup Lamiales species (Table S2) on sequence alignments in two ways; one on the complete chloroplast genome sequences, and the other on 75 protein-coding genes. *Lactuca sativa* (Asteraceae) was used as the outgroup. First, sequences were aligned using MAFFT v7.0 (http://mafft.cbrc.jp/alignment/server/) [26]. Then, jModelTest2 implemented on XSEDE (2.1.6) at the CIPRES Science Gateway (http://www.phylo.org/) was used to select the best model for maximum likelihood (ML) and standard Bayesian inference (BI) analysis. ML analysis was implemented using RAxML-HPC2 on XSEDE (8.2.10) based on the GTR + G + I nucleotide substitution model as recommended by jModelTest2 with 1000 replications. Similarly, BI analysis was constructed by MrBayes on XSEDE (3.2.6) based on the GTR + G + I nucleotide substitution model. Two independent Markov chain Monte Carlo (MCMC) chains were run for 10,000,000 generations and sampled every 1000 generations with the first 25% of calculated trees was discarded as burn-in. All the generated trees were modified by Interactive Tree Of Life (iTOL, http://itol.embl.de/) [27].

## 3. Results

### 3.1. Characteristics of the Chloroplast Genomes

**Figure 1:**
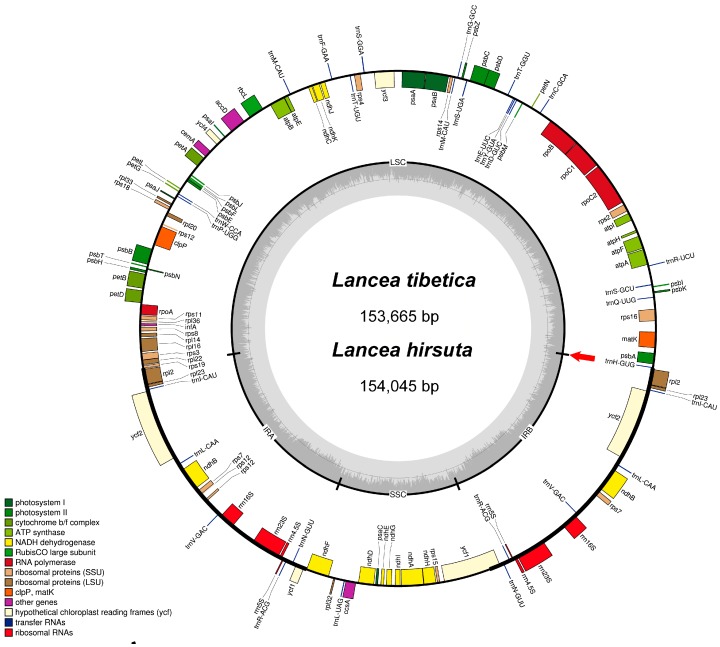
Gene map of the two *Lancea* chloroplast genomes. Genes belonging to different functional groups are color-coded. Genes drawn inside the circle are transcribed clockwise, while outside are counterclockwise. Nucleotide position 1 was indicated by the red arrow and the sequence was in counterclockwise. Gene *trnT-UGU* was not found in *L. hirsuta.*

The *L*. *tibetica* and *L*. *hirsuta* chloroplast genomes were 153,655 bp and 154,045 bp in length, respectively. The genomes were like those of most angiosperms with a typical quadripartite structure consisting of a pair of inverted repeats (IRs) of 25,624 bp in *L. tibetica* and 25,838 bp in *L. hirsuta*, a large single copy (LSC) region of 84,401 bp in *L. tibetica* and 84,588 bp in *L. hirsuta*, and a small single copy (SSC) region of 18,016 bp in *L. tibetica* and 17,781 bp in *L. hirsuta* (Figure 1, Table 1). The GC content of the genomes were both 37.9%, but the IR regions had higher GC contents (43.3% and 43.2% in *L. tibetica* and *L. hirsuta*, respectively) than that of the LSC regions (35.9% and 35.8% in *L. tibetica* and *L. hirsuta*, respectively) and SSC regions (30% and 32% in *L. tibetica* and *L. hirsuta*, respectively).

The gene content, order, and orientation were similar across the two *Lancea* genomes. There were 106 predicted genes in *L. tibetica* including 79 protein-coding genes, 23 tRNA genes, and 4 rRNA genes, while the 105 genes predicted in *L. hirsuta* consisted of 79 protein-coding genes, 22 tRNA genes, and 4 rRNA genes (Table 2). Among the protein-coding genes, 63 were located in the LSC region, 11 were in the SSC region, and 6 genes (*ndhB*, *rpl2*, *rpl23*, *rps7* and *ycf2*) were duplicated in the IR regions. There were 13 intron-containing protein-coding genes, two of which (*ycf3* and *clpP*) contained two introns. As in most other land plants, the *rps12* gene was a trans-spliced gene, with its 5′ end located in the LSC region and its duplicated 3′ ends in the IR regions. The *ndhD* gene contained the alternative ACG start codon, while *rps19* started with GTG, which are common features of most homologous genes in the chloroplast genomes of other plants [28,29,30,31,32]. Approximately 54.8% of *Lancea* chloroplast genomes consisted of protein-coding genes (84,254 bp in *L. tibetica* and 84,474 bp in *L. hirsuta*), 1.4% of tRNAs (2198 bp *L. tibetica* and 2131 bp in *L. hirsuta*), and 6.1% of rRNAs (9396 bp in both species). As such, the non-coding regions consisting of introns, pseudogenes, and intergenic spacers accounted for 37.7% of the both genomes.

### 3.2. Repeat and SSR Analysis

**Figure 2:**
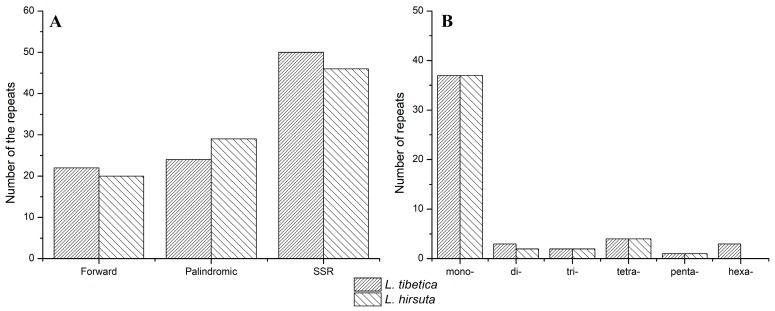
Repeated sequences in *Lancea* chloroplast genomes. (**A**) Number of three repeat types within chloroplast genomes; (**B**) SSR type distribution within *Lancea* chloroplast genome.

Total of 22 forward repeats and 24 palindromic repeats were found in the *L. tibetica* genome, while there were 20 forward repeats and 28 palindromic repeats in *L. hirsuta* (Figure 2A, Tables S3 and S4). The majority of repeats ranged in size from 30 to 44 bp, and the longest palindromic repeat (410 bp) was found in *L. hirsuta*. The repeats were mostly distributed in the intergenic spacers (IGS) and intron sequences, but eight repeats were also found in the coding sequences (CDSs) of *psaB*, *ycf3*, *rps19*, *ndhB*, *ndhA* and *ycf2*.

Simple sequence repeats (SSRs) are another important type of repeated sequence in genomes that are particularly useful molecular markers in genetic diversity research. A total of 50 perfect microsatellites, 574 bp in length, were detected in *L. tibetica* chloroplast genome, and there were 37, 3, 2, 4, 1, and 3 mono-, di-, tri-, tetra-, penta- and hexa-nucleotides repeats, respectively (Table S5). In *L. hirsuta*, the 46 SSRs with totally 496 bp in length included 37, 2, 2, 4, and 1 mono-, di-, tri-, tetra-, and penta-nucleotide repeats, respectively (Table S6). No hexa-nucleotide repeats were found in *L. hirsuta*. Most SSRs were located in non-coding regions, especially in the LSC region. AT content comprised 86% and 87% of SSRs in *L. tibetica* and *L. hirsuta*, respectively.

### 3.3. IR Contraction and Expansion

**Figure 3:**
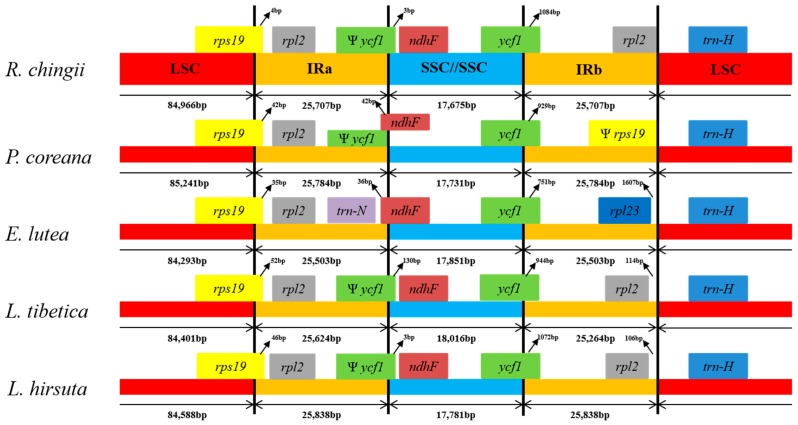
Comparison of the borders of large single-copy (LSC), small single-copy (SSC), and inverted repeat (IR) regions among the chloroplast genomes of five species.

Contraction and expansion at the borders of IR regions have been commonly reported in chloroplast genomes, which may explain the apparent size differences between chloroplast genomes [17]. Accordingly, the inferred assembly was checked to confirm contraction and expansion. Although the IR region of the five chloroplast genomes was highly conserved, structure variation was still found in the IR/SC boundary regions. As shown in Figure 3, the *rps19-rp12* gene was located in the junctions of the LSC/IRb regions. The *rps19* gene crossed the LSC/IRb region with 3–52 bp located in the IRb region. The *ycf1-ndhF* gene was located at the junctions of the IRb/SSC regions, though the *trnN-ndhF* sequence in *E. lutea* was missing the *ycf1* gene in the IRb region. The *ycf1* genes of *R. chingii*, *L. tibetica*, and *L. hirsuta* spanned the IRb and SSC regions, with 3–130 bp in the SSC region. The *ndhF* gene in *P. coreana* extended into the LSC region and overlapped with the *ycf1* gene by 42 bp, while in *E. lutea* it extended 36 bp into the LSC region. The SSC/IRa junctions in all five chloroplast genomes were crossed by *ycf1*, with 751–1084 bp in the IRa region. Like the IRb/SSC boundary regions, the LSC/IRa regions were also variable. The *rpl2-trnH* genes of *R. chingii*, *L. tibetica*, and *L. hirsuta* were located at the junctions of IRa/LSC regions with 0, 114, and 106 bp, respectively, separating the spacer from the ends of the IRa regions. However, in *P. coreana*, the *rps19* pseudogene was at one end of the IRa region. In *E. lutea*, the *rpl2* gene was missing, and *rpl23* was the last gene, with 1607 bp between the spacer and the ends of the IRa regions. Overall, contraction and expansion of the IR regions was detected across the five chloroplast genomes.

### 3.4. Sequence Divergence and Divergence Hotspot

**Figure 4:**
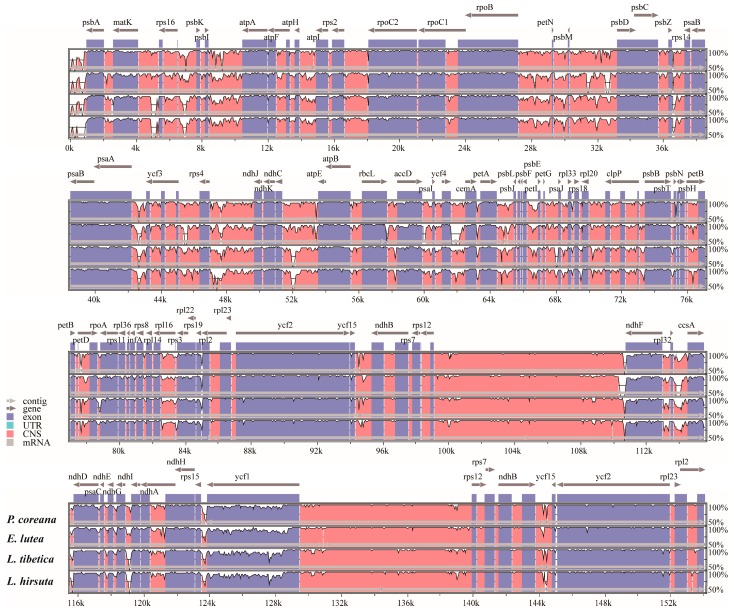
Comparison of five chloroplast genomes using the mVISTA alignment program with *Rehmannia chingii* as a reference. The *x*-axis represents the coordinates in the chloroplast genome. The *y*-axis indicates the average percent identity of sequence similarity in the aligned regions, ranging between 50% and 100%. Genome regions are color coded as protein coding, rRNA coding, tRNA coding or conserved noncoding sequences (CNS).

To characterize genome divergence, we performed multiple sequence alignments between the five chloroplast genomes using the program mVISTA, with *R. chingii* being used as a reference (Figure 4). The comparison demonstrated that the coding regions are more conserved than the non-coding regions. In particular, the IR regions were less divergent than the LSC and SSC regions. The most highly divergent regions among the five chloroplast genomes were found among the intergenic spacers, including *trnH-psbA*, *matk-rps16*, *rps16-psbK petN-psbM*, *psbZ-rps14*, *psaA-ycf3*, *rps4-ndhJ*, *ndhC-atpE*, *petA-psbJ*, and *ycf4-cemA* in LSC as well as *rpl32-ccsA* and *ndhG-ndhI* in SSC. More divergence of coding regions was found in the *matK*, *rpoA*, *rps19*, *ndhF*, *ccsA*, *ndhD*, and *ycf1* sequences. Similar results have been observed in previous studies [29,32].

**Figure 5:**
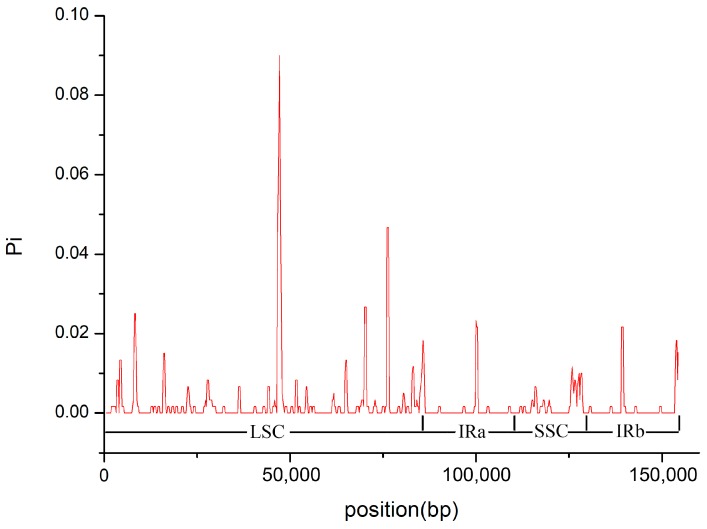
Sliding window analysis of nucleotide variability (pairwise divergence) between *Lancea tibetica* and *L*. *hirsuta*.

Nucleotide variability (pairwise divergence) was calculated to show divergence at the sequence level between the two *Lancea* chloroplast genomes. Between *L. tibetica* and *L. hirsuta*, the pairwise divergence values ranged from 0 to 0.09, with a mean of 0.00221. As shown in Figure 5, the IR regions were more conserved than the LSC and SSC regions. The most divergent region, *rps4-ndhJ*, showed a pairwise divergence value of 0.09 in the LSC region. The *petB* gene in the LSC region showed the highest degree of nucleotide variability, with a pairwise divergence of 0.0467. The low divergence values between the *L. tibetica* and *L. hirsuta* chloroplast genomes illustrated high similarity between the two species.

### 3.5. Phylogenomic Analysis

**Figure 6:**
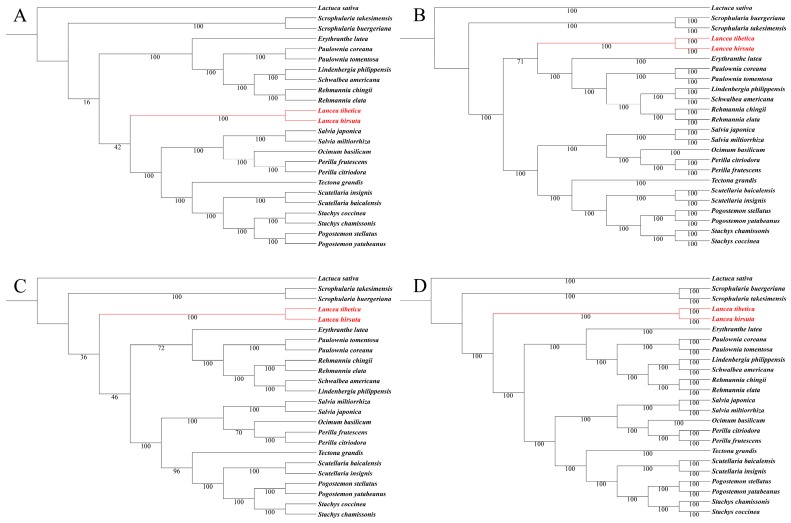
Phylogenetic trees of 24 species based on complete chloroplast genomes and 75 protein-coding genes. (**A**) Maximum likelihood (ML) phylogenetic tree constructed with complete chloroplast genomes; (**B**) Bayesian inference (BI) phylogenetic tree constructed with complete chloroplast genomes; (**C**) ML phylogenetic tree constructed with 75 protein-coding genes; (**D**) BI phylogenetic tree constructed with 75 protein-coding genes. The *Lancea* species are shown in red.

*Lancea* was traditionally placed in Scrophulariaceae. Recent studies have reported its phylogenetic relationship among other genera in the Lamiales based on chloroplast and/or nuclear ribosomal sequence data. However, the position of *Lancea* was still unclear, and thus required confirmation with additional data. In our present studies, the ML and BI analysis of the complete chloroplast genomes and 75 protein-coding genes showed that the two *Lancea* species were clustered into one monophyletic group (Figure 6). Alignment of the complete chloroplast genome sequences gave an obvious conflict between ML phylogenetic trees and BI phylogenetic trees, which may be caused by rapidly evolving and potentially poorly aligned sites [4,33,34]. On the other hand, alignment of 75 protein-coding genes strongly supported the *Lancea* genus as sister to a clade formed by Phrymaceae, Paulowniaceae, and Rehmanniaceae, rather than the Scrophulariaceae clade both in ML and BI phylogenetic trees. The relationships supported by our analysis are basically consistent with APG IV [14]. As there is a lack of published chloroplast genomes from the *Mazus*, *Dodartia*, and Phrymaceae taxa, the phylogenetic placement of Mazaceae and Phrymaceae remains uncertain.

## 4. Discussion

Using next-generation sequencing data, two complete *Lancea* chloroplast genomes were assembled, annotated, and analyzed. In the future, we plan to analyze chloroplast genomes of the genera *Mazus* and *Dodartia,* which were placed in Mazaceae in APG IV [14], in order to elucidate the phylogenetic relationships between *Lancea* and those species. Hence, the comprehensive data presented in this study not only characterizes the entire *Lancea* chloroplast genomes and enables the inference of their phylogenetic relationships, but also offers a valuable resource for future studies.

## Figures and Tables

**Table 1:** The basic chloroplast genome characteristics of *Lancea tibetica* and *L. hirsuta.*

Characteristics	*L. tibetica*	*L. hirsuta*
Total cpDNA size (bp)	153,664	154,045
Length of large single copy (LSC) region	84,401	84,254
Length of inverted repeat (IR) region	25,624	25,838
Length of small single copy (SSC) region	18,016	17,781
Total GC content (%)	37.9	37.9
LSC	35.9	35.8
IR	43.3	43.2
SSC	30.0	32.0
Total number of genes	106	105
Protein-coding genes	79	79
rRNAs genes	4	4
tRNAs genes	23	22

**Table 2:** Genes present in *Lancea tibetica* and *L. hirsuta* chloroplast genomes.

Category	Name
Rubisco	*rbcL*
Photosystem I	*psaA*, *B*, *C*, *I*, *J*
Photosystem II	*psbA*, *B*, *C*, *D*, *E*, *F*, *H*, *I*, *J*, *K*, *L*, *M*, *N*, *T*, *Z*
ATP synthase	*atpA*, *B*, *E*, * *F*, *H*, *I*
Cytochrome b/f complex	*petA*, * *B*, * *D*, *G*, *L*, *N*
Cytochrome c synthesis	*ccsA*
NADPH dehydrogenase	* *ndhA*, *^,a^ *B*, *C*, *D*, *E*, *F*, *G*, *H*, *I*, *J*, *K*
Transcription	*rpoA*, *B*, * *C1*, *C2*
Small subunit ribosomal proteins	*rps2*, *3*, *4*, *^a^ 7*, *8*, *11*, ** *12*, *14*, *15*, * *16*, *18*, *19*,
Large subunit ribosomal proteins	*^,a^ *rpl2*, *14*, * *16*, *20*, *22*, ^a^ *23*, *32 33*, *36*
Translation initiation factor	*infA*
Ribosomal RNA	^a^ *rrn4.5*, ^a^ *5*, ^a^ *16*, ^a^ *23*
RNA processing	*matK*
Carbon metabolism	*cemA*
Fatty acid synthesis	*accD*
Proteolysis	** *c1pP*
Unknown function protein-coding gene	*ycf1*, *2*, ** *3*, *4*
Transfer RNA	*trnC-GCA*, *trnD-GUC*, *trnE-UUC*, *trnF-GAA*, *trnG-GCC*, *trnH-GUG*, ^a^ *trnI-CAU*, ^a^ *trnL-CAA*, *trnL-UAG*, ^a^ *trnM-CAU*, ^a^ *trnN-GUU*, *trnP-UGG*, *trnQ-UUG*, ^a^ *trnR-ACG*, *trnR-UCU*, *trnS-GCU*, *trnS-GGA*, *trnS-UGA*, *trnT-GGU*, ^b^ *trnT-UGU*, ^a^ *trnV-GAC*, *trnW-CCA*, *trnY-GUA*

* gene with one intron, ** gene with two introns, ^a^ gene with two copies, ^b^ gene not found in *L. hirsuta*.

## Supplementary Materials

Supplementary materials are available online. Table S1: List of all pairs of primers used for assembly validation; Table S2: The list of accession numbers of the chloroplast genome sequences used in the phylogenetic analysis; Table S3: Long repeat sequences in the *Lancea tibetica* chloroplast genome; Table S4: Long repeat sequences in the *Lancea hirsuta* chloroplast genome; Table S5: Distribution of SSRs in the *Lancea tibetica* chloroplast genome; Table S6: Distribution of SSRs in the *Lancea hirsuta* chloroplast genome.
