# Supplementary material for: The Complete Chloroplast Genomes of Two Lancea Species with Comparative Analysis
Source: Molecules. 2018 Mar 7;23(3):602. doi: 10.3390/molecules23030602 (PMC6017492; doi:10.3390/molecules23030602)
Supplement: Supplementary file 1 [file molecules-23-00602-s001.pdf]

Supplementary Material

Table S1. List of all pairs of primers used for assembly validation.

Taxon	Regions	Sequence (5'-3')
L. tibetica	LSC/IRa	F: TGGTTGACGCCACAAATTCC
		R: CGGACCATCCATAGCAGTCA
	IRa/SSC	F: AATTCCATCCCCACAAACCGT
		R: GCCATTTCAAGTCTTGCTCCC
	SSC/IRb	F: GGTAATCTCTCACACTCGGCT
		R: ATCGGACCATCCATAGCAGTC
	IRb/LSC	F:TTCCATCCCCACAAACCGT
		R: AGTCTTGCTCCCATTGGACTT
L. hirsuta	LSC/IRa	F: CCTAAAGCGCGTACTTCCGT
		R: GATCCAAGCGTTGGCTAGGT
	IRa/SSC	F: GGAACAAGAGGGATCCACCG
		R: CAACATCCTTTGTTGGGGCG
	SSC/IRb	F: TTTTCAAGTGTAGGCGGGGA
		R: GGCAGAATACCGTCACCCAT
	IRb/LSC	F: AACCCTGTAGACCATCCCCA
		R: CCTAGCTGCTTGGCCTGTAG

Table S2. The list of accession numbers of the chloroplast genome sequences used in the phylogenetic analysis.

**No.**	**Taxon**	**Family**	**GenBank Accession Number**
**1**	*Erythranthe lutea*	Phrymaceae	NC_030212.1
**2**	*Lindenbergia philippensis*	Orobanchaceae	NC_022859.1
**3**	*Ocimum basilicum*	Labiatae	NC_035143.1
**4**	*Paulownia coreana*	Paulowniaceae	NC_031435.1
**5**	*Paulownia tomentosa*	Paulowniaceae	NC_031436.1
**6**	*Perilla citriodora*	Labiatae	NC_030755.1
**7**	*Perilla frutescens*	Labiatae	NC_030756.1
**8**	*Pogostemon stellatus*	Labiatae	NC_031434.1
**9**	*Pogostemon yatabeanus*	Labiatae	NC_031433.1
**10**	*Rehmannia chingii*	Orobanchaceae	NC_033534.1
**11**	*Rehmannia elata*	Orobanchaceae	NC_034312.1
**12**	*Salvia japonica*	Labiatae	NC_035233.1
**13**	*Salvia miltiorrhiza*	Labiatae	NC_020431.1
**14**	*Schwalbea americana*	Orobanchaceae	NC_023115.1
**15**	*Scrophularia buergeriana*	Scrophulariaceae	NC_031437.1
**16**	*Scrophularia takesimensis*	Scrophulariaceae	NC_026202.1_
**17**	*Scutellaria baicalensis*	Labiatae	NC_027262.1
**18**	*Scutellaria insignis*	Labiatae	NC_028533.1
**19**	*Stachys chamissonis*	Labiatae	NC_029822.1
**20**	*Stachys coccinea*	Labiatae	NC_029823.1
**21**	*Tectona grandis*	Verbenaceae	NC_020098.1

**22**	*Lactuca sativa*	Asteraceae	NC_007578.1

Table S3. Long repeat sequences in the *Lancea tibetica* chloroplast genome.

**No.**	**size**	**type**	**Repeat 1 Start**	**Repeat 2 Start**	**E-Value**
**1**	58	F	47369	47397	7.99E-26
**2**	56	F	69770	69823	1.28E-24
**3**	39	F	43684	119893	2.20E-14
**4**	41	F	98636	119891	1.69E-13
**5**	39	F	43684	98638	2.57E-12
**6**	37	F	91642	91660	2.11E-09
**7**	37	F	146369	146387	2.11E-09
**8**	30	F	47369	47425	5.76E-09
**9**	37	F	91622	91640	7.38E-08
**10**	37	F	146389	146407	7.38E-08
**11**	35	F	43687	95598	9.94E-07
**12**	35	F	91624	91660	9.94E-07
**13**	35	F	95598	119896	9.94E-07
**14**	35	F	146371	146407	9.94E-07
**15**	34	F	7910	35530	3.64E-06
**16**	34	F	16082	16083	3.64E-06
**17**	34	F	91652	91670	3.64E-06
**18**	32	F	146397	146415	4.82E-05
**19**	30	F	9492	36500	6.31E-04
**20**	30	F	37847	40083	6.31E-04
**21**	30	F	89222	89264	6.31E-04
**22**	30	F	148772	148814	6.31E-04
**23**	41	P	119891	139389	1.69E-13
**24**	44	P	74812	74812	1.83E-13
**25**	39	P	43684	139389	2.57E-12
**26**	39	P	60029	60029	2.57E-12
**27**	32	P	9074	9074	3.60E-10
**28**	38	P	59630	59630	5.56E-10
**29**	37	P	91642	146369	2.11E-09
**30**	37	P	91660	146387	2.11E-09
**31**	30	P	7917	45435	5.76E-09
**32**	37	P	91622	146389	7.38E-08
**33**	37	P	91640	146407	7.38E-08
**34**	35	P	43687	142433	9.94E-07
**35**	35	P	91624	146371	9.94E-07
**36**	35	P	91660	146407	9.94E-07
**37**	35	P	119896	142433	9.94E-07
**38**	34	P	91652	146362	3.64E-06
**39**	34	P	91670	146380	3.64E-06
**40**	31	P	43684	76122	1.75E-04
**41**	31	P	55138	66463	1.75E-04
**42**	31	P	76122	119893	1.75E-04
**43**	30	P	35537	45435	6.31E-04
**44**	30	P	89222	148772	6.31E-04
**45**	30	P	89264	148814	6.31E-04

Table S4. Long repeat sequences in the *Lancea hirsuta* chloroplast genome.

**No.**	**size**	**type**	**Repeat 1 Start**	**Repeat 2 Start**	**E-Value**
**1**	62	F	77809	77870	3.13E-28
**2**	44	F	70333	70381	2.15E-17
**3**	39	F	44334	120717	2.20E-14
**4**	38	F	63264	63301	8.80E-14
**5**	41	F	94900	94938	1.69E-13

**6**	41	F	99395	120715	1.69E-13
**7**	41	F	144714	144752	1.69E-13
**8**	39	F	44334	99397	2.57E-12
**9**	39	F	111669	111702	1.47E-10
**10**	32	F	43766	43797	3.60E-10
**11**	37	F	92363	92381	2.11E-09
**12**	37	F	147275	147293	2.11E-09
**13**	32	F	76476	139897	3.46E-08
**14**	37	F	92343	92361	7.38E-08
**15**	37	F	147295	147313	7.38E-08
**16**	34	F	100291	100316	1.14E-07
**17**	34	F	139343	139368	1.14E-07
**18**	31	F	61923	61954	1.34E-07
**19**	35	F	44337	96357	9.95E-07
**20**	35	F	92345	92381	9.95E-07
**21**	410	P	196	85364	2.93E-229
**22**	389	P	287	85294	1.10E-216
**23**	381	P	295	85294	1.79E-214
**24**	347	P	329	85294	8.42E-197
**25**	139	P	15	85810	1.37E-74
**26**	109	P	771	85141	1.58E-56
**27**	100	P	186	85684	1.81E-44
**28**	69	P	607	85294	1.91E-32
**29**	59	P	732	85232	3.08E-22
**30**	41	P	94900	144714	1.69E-13
**31**	41	P	94938	144752	1.69E-13
**32**	41	P	120715	140257	1.69E-13
**33**	44	P	75421	75421	1.83E-13
**34**	39	P	44334	140257	2.57E-12
**35**	39	P	60433	60433	2.57E-12
**36**	37	P	156	85777	3.91E-11
**37**	32	P	9691	9691	3.60E-10
**38**	38	P	60034	60034	5.57E-10
**39**	37	P	92363	147275	2.11E-09
**40**	37	P	92381	147293	2.11E-09
**41**	30	P	8536	46043	5.76E-09
**42**	32	P	76476	99764	3.46E-08
**43**	37	P	92343	147295	7.38E-08
**44**	37	P	92361	147313	7.38E-08
**45**	34	P	100291	139343	1.14E-07
**46**	34	P	100316	139368	1.14E-07
**47**	35	P	44337	143301	9.95E-07
**49**	35	P	92345	147277	9.95E-07

Table S5. Distribution of SSRs in the *Lancea tibetica* chloroplast genome.

Nucleotide	Total Counts	Total Length(bp)	Average Length(bp)	Frequency(loci/Mb)	Density(bp/Mb)
**mononucleotide**	**37**	**403**	**10.89**	**240.78**	**2622.588**
**dinucleotide**	**3**	**30**	**10**	**19.52**	**195.23**
**trinucleotide**	**2**	**24**	**12**	**13.02**	**156.184**
**tetranucleotide**	**4**	**48**	**12**	**26.03**	**312.368**
**pentanucleotide**	**1**	**15**	**15**	**6.51**	**97.615**
**hexanucleotide**	**3**	**54**	**18**	**19.52**	**351.414**

Table S6. Distribution of SSRs in the *Lancea hirsuta* chloroplast genome.

Nucleotide	Total Counts	Total Length(bp)	Average Length(bp)	Frequency(loci/Mb)	Density(bp/Mb)
**mononucleotide**	37	389	10.51	240.6	2529.588
**dinucleotide**	2	20	10	13.01	130.056
**trinucleotide**	2	24	12	13.01	156.067
**tetranucleotide**	4	48	12	26.01	312.134
**pentanucleotide**	1	15	15	6.5	97.542
